# Health and educational achievement of school-aged children: The impact of anaemia and iron status on learning

**DOI:** 10.4102/hsag.v24i0.1101

**Published:** 2019-05-20

**Authors:** Thando P. Gwetu, Myra Taylor, Meera Chhagan, Shuaib Kauchali, Murray Craib

**Affiliations:** 1 Discipline of Public Health, University of KwaZulu-Natal, Durban, South Africa; 2 Department of Paediatrics and Child Health, University of KwaZulu-Natal, Durban, South Africa

**Keywords:** anaemia, iron status, growth, development, school performance

## Abstract

**Background:**

Anaemia is a common blood disorder in children and is known to cause complications such as lethargy and stress on bodily organs. Children from disadvantaged communities often fail to achieve their age-related potential with iron deficiency anaemia stated as a risk factor through causing inattentiveness and learning problems. Limited evidence exists for the adverse effects of iron deficiency anaemia on the developing child’s brain from South African studies.

**Aim:**

The objective of this study was to determine the local prevalence of anaemia and iron deficiency and to examine their association with psychomotor development and school performance in school-aged children.

**Setting:**

This study was conducted in a peri-urban disadvantaged community from KwaZulu-Natal, South Africa.

**Methods:**

Children aged 6 to 8 years from KwaZulu-Natal were enrolled (*n* = 184). Three parameters of assessment were used: clinical data, biochemical data (haemoglobin levels and iron studies) and school performance (interviews with caregivers, teachers and the children). Anaemia presence and iron deficiency were the hypothesised mediating variables through which growth, development and school performance were influenced.

**Results:**

A high point prevalence of anaemia (23.4%), iron deficiency anaemia (4.9%) and helminth infection (27.1%) was identified. Impaired cognitive assessment scores (20.7%) were prevalent in the children sampled. Behavioural problems (4.3%), poor memory function (4.3%) and impaired attention (1.1%) were of low prevalence. Anaemia and iron deficiency were both associated with impaired fine motor skills (*p* < 0.05). Anaemia was significantly associated with low cognitive scores (*p* = 0.01). Neither anaemia, iron status nor helminth infection significantly predicted school performance in the children sampled.

**Conclusions:**

The point prevalence of anaemia and iron deficiency among the sampled children was higher than the national prevalence. The sample size was however inadequate for drawing statistical conclusions about psychomotor development and school performance because of the low prevalence of the different outcomes that were examined. Practical challenges faced in conducting this investigation in rural South African schools were discussed.

## Introduction

Attention and effort are increasingly being directed towards strategies to improve educational quality in poorly resourced schools within developing nations. Programme success has been widely reported for improved access to education. Nonetheless, concern continues regarding educational accomplishment and school completion in deprived communities (Engle et al. 2007). Poor school achievement and completion rates have been attributed to various factors inclusive of ill health and malnutrition that affect intellectual functioning and therefore the ability to benefit from schooling (Aboussaleh et al. 2009; Dissanayake et al. 2009; Jukes 2005; Olson et al. 2009). Gross motor skills affect school readiness outcomes in disadvantaged South African children (Sherry & Draper 2013). It is generally acknowledged that both marginal and deficient intakes of iron are widespread globally. Iron deficiency anaemia (IDA) is linked with fatigue, depressed mood, reduced concentration and impaired memory (Beard & Connor 2003).

Few South African studies have analysed the burden of anaemia or iron deficiency in school-aged children at national or community levels (Hall et al. 2001; Hlatshwayo 2011; Kruger et al. 2014). The South African National Health and Nutrition Examination Survey (SANHANES-1) conducted in 2012 reported a national anaemia prevalence of 10.5% and iron deficiency of 11% for children aged up to 14 years (Shisana et al. 2013). The investigational evidence relating the influence of IDA or non-iron deficiency anaemia (NIDA) on psychomotor development, cognitive abilities or the impact on intellect is poor. Few studies have reported on ways for schools to approach the challenge of anaemia among schoolchildren (Hall et al. 2001). This study expects to contribute to the health and education discussion by describing the impact of anaemia and iron deficiency on psychomotor development and school performance among school-aged children from a peri-urban disadvantaged community.

## Methods

### Study design

This cross-sectional study employed a descriptive and analytical approach using observation, interviews, clinical assessments and laboratory testing. An exploratory–descriptive research method was utilised to gain insight into the area of school performance and child psychomotor development differences in the school-aged children with anaemia and iron deficiency, as there was limited research and information in this area.

This study was an ancillary study to the main Asenze study, which was an epidemiological cohort study investigating the health and psychosocial needs of children. The main study enrolled 1787 children aged between 4 and 6 years. During the main study baseline clinical assessments were conducted on all the children, and participants who were identified with anaemia, malnutrition or other illnesses were given nutritional counselling and referred for further management to appropriate health and social services’ facilities. This current study was conducted during the second phase of the main study, when these children were being followed up after school entry at the age of 6 to 8 years.

### Study setting and population

This study was conducted in a peri-urban disadvantaged community from KwaZulu-Natal, South Africa. This setting was chosen for several reasons: high anaemia prevalence (Shisana et al. 2013), high helminth infestation (Saathoff et al. 2004) and quintiles 1 and 2 schools providing low educational resources (KwaZulu-Natal Department of Education 2011). Education in the area is among the most deprived in South Africa. A report by the KwaZulu-Natal Department of Education on literacy and numeracy indicated that an average of 62% of grades 3 and 6 learners did not have basic literacy and numeracy proficiency (KwaZulu-Natal Department of Education 2011).

A statistician was consulted to assist with the sample size determination. An anaemia prevalence of 21.1% (WHO 2008) was considered representative of the sample population. The study population was randomly selected and could be reasonably expected to represent the general local population, in which low socio-economic status and under-nutrition prevail, given the high prevalence of growth stunting in South African children (Kruger et al. 2014) and low cognitive scores (Grover raw scores) of 48% (interquartile range [IQR] 27, 56) from a pilot study by the main study, not yet adjusted for age or other factors.

### Data collection

The components of this assessment comprised both information collection and clinical assessment. This research used three parameters of assessment: clinical data, biochemical data and school performance.

Clinical data: All participants received a comprehensive medical assessment of health, physical functioning and cognitive functioning, which were conducted by a trained medical officer from the main study (Table 1).

**TABLE 1:** Clinical assessments performed on the sample population by a trained research clinician.

Information collection	History of child’s health and growth
Anthropometric parameters	Current weight and height Use of standard World Health Organization growth charts
General examination	Features of congenital abnormalities or disabilities
Systemic examination	Head and neck, chest, abdomen, genitourinary, musculoskeletal, skin, neurological
Other assessments	Vision (Snellen chart), hearing (tympanometry), oral health

Biochemical data: The principal investigator (T.P.G.) collected all the blood samples, which were analysed at a local accredited laboratory. Venous blood samples were collected for haemoglobin (Hb) levels, serum ferritin, C-reactive protein and soluble transferrin receptor. These biochemical studies for iron status were used to assess the nature of anaemia, in terms of presence of iron deficiency, and hence distinguish IDA from NIDA. Anaemia was defined using Hb levels and was classified as mild for Hb levels 11.0 g/dL−11.4 g/dL, moderate for levels 8.0 g/dL−10.9 g/dL and severe at Hb <8.0 g/dL (WHO 2011). C-reactive protein was used as a general marker for inflammation and infection and hence as a very rough proxy for NIDA. The estimation of the body iron (mg/kg body weight) was described in a separate unpublished journal article, which is under review. Stool and urine samples were also collected, stored in a refrigerator and sent within 24−48 h for microscopy and detection of parasite infection at a local academic laboratory. The results of the participants’ HIV infection status were available from the main study. Malaria infection screening was not conducted as the study area was in a non-malaria endemic region.

School achievement: In-depth interviews were conducted with caregivers, teachers and the children. The caregiver was questioned about the child’s performance and behaviour at school. Teacher interviews about the child’s progress were conducted at the child’s school. Teacher strengths and difficulties questionnaires were administered during interviews conducted by research nurses with the children’s teachers to assess school achievement and behaviour in comparison to other children in the class. The assessed academic subjects in the schools visited were IsiZulu, English, Numeracy and Life Skills. The child’s last school report was also analysed. This study did not assess the school’s teaching quality or book procurement. The study focused on previous attainments of the children using earlier school assessments.

Cognitive and behavioural development: Children’s intellectual capacities were assessed using a range of tools such as interviewer-based schedules, observational checklists and behaviour rating scales. The skills assessed were global cognitive, specific cognitive, social communication, memory, attention, visuospatial, reading, numeracy and motor skills (Table 2). The International Classification of Functioning, Disability and Health (ICF) (WHO 2001) was used to describe the health profiles of each participant’s functioning, disability and health status in various domains such as motor skills, vision, hearing, speech, behavioural and cognitive development.

**TABLE 2:** Cognitive and behavioural assessments performed on the sample population.

Variable	Description
Gross motor development	The skills were classified into three groups: Locomotor – run, gallop, hop, leap, jump and slide. Body manipulation – stretch, curl, twist, roll, bend and balancing skills. Object control – throw, catch, strike, kick and ball bouncing. The assessment involved use of a checklist with skills scored pass or fail and individual performances classified as normal, suspect or delayed.
Fine motor assessment	Skills were classified into two groups: Classroom skills such as cutting and writing. Supporting skills such as crossing midline and hand strength.
Vision	Visual acuity, contrast sensitivity and colour vision were assessed using a psychophysical method of limits test with yes or no responses.
Hearing and speech	Assessed using case history, physical exam, tests of middle ear function, pure-tone audiometry, speech audiometry, receptive and expressive language.
Cognitive and behavioural tests	A range of tools were used such as interviewer-based schedules, observational checklists and behaviour rating scales. Skills assessed were global cognitive, specific cognitive, social communication, memory, attention, visuospatial, reading, numeracy and motor skills.

### Validity and Reliability

The questionnaires were pre-tested in the research area. Ten participants were interviewed. The same ten persons were relocated again for the assessment of the test-retest reliability. The participants were asked to give an opinion of the interview and the time spent was recorded. The questionnaires were adjusted according to feedback from the participants and the initial findings of shortcomings in the questionnaire content.

### Data analysis

Data were entered and analysed using SPSS version 23 for Windows. Children who had anaemia or iron deficiency during the study were included in the case group. The level of significance was *p* < 0.05. Differences between proportions were considered statistically significant if the 95% confidence interval (CI) did not overlap between anaemic groups compared to the control population in the parameters assessed such as cognitive function, behaviour and school performance. Chi-square or Fisher’s exact tests were used for contingency table data to assess the relationship between anaemia and categorical variables in order to determine the difference between the anaemic and normal group in school performance. The *t-*test was used to compare the means and standard deviation for continuous variables such as age and cognitive scores. Linear regression models were conducted to quantify the relationship between school performance and both anaemia presence and iron status. The distributions were also assessed graphically. Previous studies have reported that specific helminth infections were related to cognitive deficits and to IDA (Jinabhai et al. 2002; Kvalsvig, Cooppan & Connolly 1991; Pabalan et al. 2018); hence, the presence of helminth infections was also analysed so as to assess whether helminth infections confounded the relationship between school performance, anaemia type and iron deficiency or if helminth infection was an independent predictor.

### Ethical considerations

The study questionnaires and blood sampling were approved under the main study by the University of KwaZulu-Natal Biomedical Research Ethics Committee (Ref no. BF036/07). School-based interviews were approved by the Department of Education (Ref. 2/4/8/1). Informed consent for this study was sought separately from that provided for the main study. Written informed consent was obtained from the caregivers of the participants before the data collection was undertaken. A child’s affirmative agreement to participate in this research was considered sufficient for assent.

When there was an identified need for consultation, assessment, intervention, therapy or resources, the child was referred to the appropriate healthcare or other professionals using local referral channels. All those with identified ill health or impairments were referred to the local clinic for appropriate management and follow-up. Feedback in the form of a confidential report for each participant was given directly to the caregivers concerned.

## Results

### Sample population

The children sampled (*n* = 184) had a mean age of 6.5 ± 0.55 years and comprised more males (109; 59.2%) than females (75; 40.8%). The difference in age between the genders was not statistically significant. All the sampled children were asymptomatic for anaemia or any other active disease. None of the children suffered with seizures or any known cognitive impairment.

### Anaemia, iron status and clinical factors

The mean Hb level for this sample was 12.17 g/dL ± 1.19 g/dL (*n* = 184). Anaemia was detected in 43 (23.4%) children (95% CI 17.8, 30.0). The calculated body iron levels were normally distributed and were 6.8 mg/kg ± 2.5 mg/kg. Of the children sampled (*n* = 184), 13 (7.1%; 95% CI 4.2, 11.7) had tissue iron depletion and of these 9/13 (69.2%; 95% CI 42.4, 87.3) were anaemic (Table 3). Referral letters for further management of anaemia at a health centre were given to 9 (48.9%) children (*n* = 184). Among the children with caregiver consent for HIV testing (*n* = 180), 5 (2.8%) tested positive for HIV infection. Anaemia was identified in 3 (60%) HIV-positive children (*n* = 5) and 1 (20%) was iron deficient. HIV-positive children had a trend towards lower Hb (11.2 g/dL ± 0.8 g/dL) than the children who were HIV negative (12.19 g/dL ± 1.19 g/dL). The differences between HIV-infected children were not extensively explored because of the small sample size, although an HIV-positive status was not associated with school performance scores (*p* = 0.18). Urine and stool samples from 181 children were available for parasite detection. Microscopy was positive in 49 (27.1%) children but only 31 (17.7%) of the children had pathologic infection. Parasitic infection was associated significantly with anaemia (*p* = 0.03), but no difference was noted with school performance. The relationship between anaemia status and the children’s anthropometric measures, diet and home environment has also been discussed in a separate paper (Gwetu et al. 2016).

**TABLE 3:** Anaemia and/or iron status prevalence.

Category	Groups	Definition	Number	Percentage	Confidence interval
Anaemia severity	Mild	Hb levels 11.0 g/dL−11.4 g/dL	24/43	55.8	38.9, 67.5
	Moderate	Hb levels 8.0 g/dL−10.9 g/dL	18/43	41.9	28.4, 56.7
	Severe	Hb < 8.0 g/dL	1/43	2.3	0.4, 12.1
Anaemia and iron status group	IDA	Anaemia and low body iron stores in the absence of inflammation	7/43	16.3	-
	NIDA	Anaemia in the presence of inflammation in a child with normal iron stores	34/43	79.1	-
	MA	Anaemia in the presence of both iron deficiency and inflammation	2/43	4.7	-
	IDS	Depleted iron stores but child was not anaemic	4/184	2.2	-
	NA	Normal haemoglobin concentrations and normal iron status	137/184	74.5	-

IDA, iron deficiency anaemia; NIDA, non-iron deficiency anaemia; MA, mixed anaemia; IDS, iron depleted stores; NA, not anaemic.

### Physical and cognitive development

#### Motor skills

The gross motor skills assessment for the sample (*n* = 184) identified one child who had an abnormal gait pattern associated with walking or running (ICF b770). The fine motor skills assessment (*n* = 184) revealed that 7 (3.8%) of the sampled children had poor control and coordination of voluntary movements (ICF b760). Another 7 (3.8%) had problems with fine motor hand use and coordinated actions associated with the manipulation of fingers and hands to handle small objects (ICF d4402). Referrals for further assessment at a health centre for developmental delay were given to 2 (1.1%) children from the study population. The presence of anaemia was significantly associated with impaired fine motor skills (*p* = 0.009) but not associated with impaired gross motor skills. Iron deficient status was also significantly associated with impaired fine motor skills (*p* = 0.023) but was not significantly associated with impaired gross motor skills. Nonetheless, because of the small numbers of children identified as having a problem with the outcome variables such as gross motor development, conclusions cannot be drawn about the relationship between motor development, anaemia and iron deficiency as this was inadequate for drawing statistical conclusions.

#### Vision, hearing and speech

The children were assessed by a medical practitioner for vision, hearing and speech (*n* = 184). Only 1 (0.5%) child had problems of visual acuity (b210.1). Assessment of hearing revealed that 16 (8.7%) of the children sampled had problems with sound discrimination (b230.1), while 2 (1.1%) had problems with location of sound source (b230.2) and 1 (0.5%) each had problems with dizziness (s240.1), sensation of falling (s240.2) and nausea associated with vertigo (s240.3). With regard to speech, 1 (0.5%) child had voice quality problems (b310.1) and 4 (2.2%) children had articulation difficulties (b320.1). Iron status was not significantly associated with hearing, vision or speech. Anaemia presence was not significantly associated with hearing, vision or speech. This study’s sample size was too small given the prevalence of these examined outcomes; hence, because of these power issues, conclusions cannot be drawn about the relationship between anaemia and IDA and these outcomes.

#### Cognitive and behavioural development

Impaired cognitive assessment scores (*n* = 184) were reported in 38 (20.7%) of the children sampled. Memory function scores (b144.1) were poor in 8 (4.3%) of these children. Thought function scores (b160.1) were low in 2 (1.1%) children and the higher-level cognitive functions score (b164.1) was low in 28 (15.2%). Behavioural problems were identified in 8 (4.3%) children, with 2 (1.1%) having impaired attention functions (b140.1). Of the children, 4 (2.2%) had poor basic learning skills in reading (b140) while 59 (32.1%) were assessed to have poor scores in the basic learning skills for numeracy (b150). One child had problems with school education and engaging in any school-related tasks; this child had been taken out of school and was currently at home. Only 1 (0.55%) child was reported to still experience urinary continence difficulties. Of the children sampled, 3 (1.6%) children were referred for further assessment and management for cognitive and behavioural performance and needs. The presence of anaemia was significantly associated with low cognitive scores (*p* = 0.01) but not with low behavioural scores. Iron deficiency was not significantly associated with low cognitive scores or behavioural scores. This study again acknowledges the low prevalence of the examined outcomes. Because of the statistical power issues, conclusions could not be drawn about the relationship between anaemia and IDA and these outcomes.

### School educational achievement

The places of education attended by the children (*n* = 184) varied, with 5 (2.7%) of the children not receiving any education at all, 3 (1.6%) attending crèche, 1 (0.5%) being in Grade R at a normal school and 175 (95.1%) being in Grade 1 or above at a normal school. The parents reported that 7 (3.8%) of the children had not yet started Grade 1 despite being of schoolgoing age and 7 (3.8%) of the children had started school but later dropped out. The place of education or child’s grade was not significantly associated with anaemia presence (*p* = 0.73) or iron deficient status (*p* = 0.36).

The class attendance (*n* = 184) for the last full term showed that 102 (55.4%) children were never absent, 32 (17.4%) missed 1–2 days, 21 (11.4%) missed 3–4 days and 10 (5.4%) missed 5–10 days. Some caregivers did not know much about the child’s school attendance (3; 1.6%) while 9 (4.9%) children were not receiving formal education. For absences in the last 4 weeks preceding the study, 142 (77.2%) children had no absenteeism, 20 (10.9%) were absent 1–2 days, 8 (4.3%) were absent 3–4 days, 1 (0.5%) was absent 5–10 days and 1 (0.5%) was unknown.

None of the children (*n* = 184) attended a special needs school and none had been considered for further educational assessment. Generally, the parents or caregivers believed that their children (*n* = 184) performed well when compared to their peers, with 2 (1.1%) who believed their children’s performance was poor, while 15 (8.2%) believed them to be average, 51 (27.7%) good, 44 (23.9%) very good and 64 (34.8%) excellent. The last school report card, however, showed that performance was not achieved with 13 (7.1%) children, partially achieved with 23 (12.5%), satisfactory for 62 (33.7%) and achieved for 71 (38.6%) of children sampled. Neither the caregiver’s perception nor the last report on school performance was significantly associated with anaemia presence or iron status.

## Teacher assessments

**FIGURE 1:**
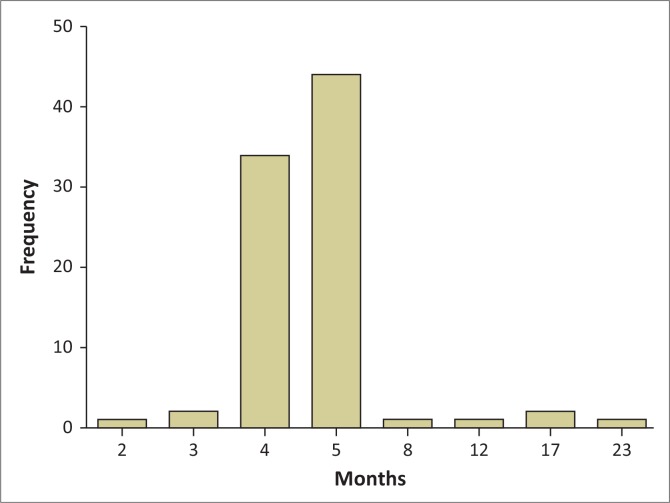
Number of months that each child had been in the teacher’s class (*n* = 184).

The teacher strengths and difficulties questionnaires (*n* = 184) were completed for only 89 (48.4%) children. Not all children had these variables assessed because their teachers were absent or the information was not available at the time the researchers went to the school; hence, more than half of the children could not be assessed for school performance by their teachers. Those children assessed (*n* = 89) attended school Grade R (1; 1.1%), Grade 1 (39; 43.8%), Grade 2 (38; 42.7%) and Grade 3 (11; 12.4%). The duration of interaction with the assessing teacher varied widely (Figure 1) and was not significantly associated with anaemia presence or iron deficiency.

**FIGURE 2:**
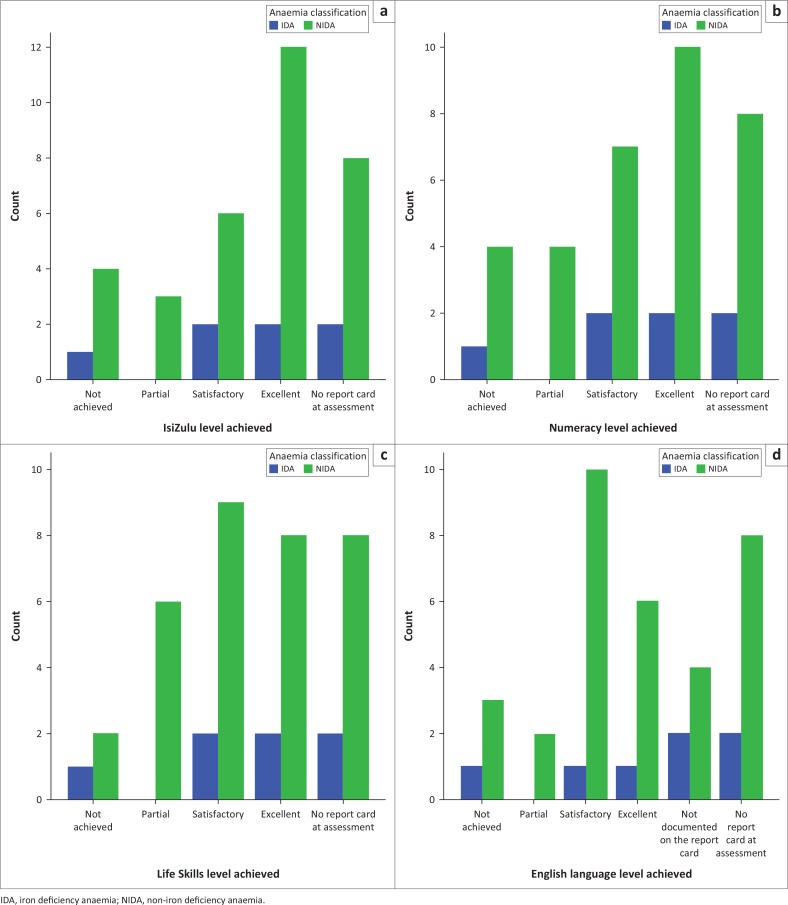
School performance based on last school report card for children with iron deficiency anaemia and non-iron deficiency anaemia (*n* = 89).

The children who were anaemic generally performed poorly compared to their non-anaemic companions in all the assessed subjects – IsiZulu, English, Numeracy and Life Skills, though this did not reach statistical significance as shown in Figure 2. A similar trend was also noted with the children who were iron deficient as they also generally performed poorly compared to their iron-replete companions in all the assessed subjects.

There was no significant difference between the school performance of children with IDA and the children with NIDA in all the assessed school subjects.

## Discussion

High levels of impaired cognitive assessment scores (20.7%) and behavioural problems (4.3%) were identified in the children sampled. This has also been noted in other studies assessing children from disadvantaged communities (Jinabhai et al. 2002; Kvalsvig et al. 1991; Pabalan et al. 2018). The presence of anaemia was significantly associated with low cognitive scores (*p* = 0.01) but did not affect the children’s behavioural scores. Anaemia was also significantly associated with impaired fine motor skills (*p* = 0.009) but not with impaired gross motor skills. The association between IDA and psychomotor development has been described by various researchers, with evidence suggesting that the adverse consequences may not be fully reversible with treatment (Hermoso et al. 2011; Wang et al. 2013).

Despite the considerable attention in epidemiological literature to the profile of NIDA, there are very few human studies that have examined the morbidity implications on cognitive status and growth. Olney et al. (2007) reported that children with NIDA had decreased cognitive performance in specific domains, compared to subjects with no anaemia. Parasitic infection was associated significantly with anaemia (*p* = 0.03) but not with school performance scores. This study measured current parasitic infection, although chronic infestation with intestinal parasites would be a better indicator in a developmental setting for factors affecting long-term intellectual development in children. Other studies have shown that helminth infection was associated with poor educational performance (Jinabhai et al. 2002; Kvalsvig et al. 1991; Pabalan et al. 2018).

In this study children with iron deficiency tended to have low cognitive and behavioural scores, although these did not reach statistical significance. Iron deficiency was significantly associated with impaired fine motor skills (*p* = 0.02) but not with gross motor skills. This was in contrast to the increasing evidence from industrialised and developing societies that suggests that a suboptimal iron status particularly if occurring over a long duration may adversely affect cognitive (Beard & Connor 2003) and motor development (Olney et al. 2007; Sachdev, Gera & Nestel 2005), as well as impair neuropsychological and social-emotional functioning and predispose children to anti-social behaviour in the short and long term (Benton 2007). The age at which iron deficiency occurred was reported to be important, with iron deficiency in early life resulting in delayed development (Beard & Connor 2003). The evidence on the effect of iron supplementation or improved diet suggests that this was ineffective if the deficiency occurred during the early years of life and over a long duration (Lozoff et al. 2000). The significance of iron deficiency occurring in preschool and older children was, however, reported to sometimes be reversible with subsequent adequate supply (Sachdev et al. 2005).

Children referred for further assessment and management at a health centre included 1.6% for cognitive and behavioural performance and needs, 1.1% of children for developmental delay and 4.9% for moderate or severe anaemia. The generally good attention function (98.9%) and memory function (95.7%) observed in this study shows good potential for enhanced school performance especially in later primary school grades (Boivin et al. 1993; Jinabhai et al. 2004). The evidence of the school identification and referral system for early intervention for learning difficulties was very weak as none of the children sampled had as yet received any recommendation for further assessment from a school teacher. This study expects that an effective school readiness assessment as well as anaemia and parasitic interventions may improve educational quality. However, limited experimental evidence exists that these interventions can work to advance educational quality (Boivin et al. 1993; Brooker et al. 2010).

A third of the sampled children were poorly equipped in the basic learning skills for numeracy. When compared to other countries South Africa fares poorly in mathematics and science and this study highlights the need to identify such problems early and to initiate remedial action for these children. Already in these early grades there is evidence that children are falling behind and the Human Sciences Research Council (HSRC) report ‘Towards equity and excellence’ highlights the gaps in the education system (HSRC 2011).

This study identified that conducting research that involved school-aged children in a rural context, and particularly in schools, raised a number of practical concerns (Aboussaleh et al. 2009; Olney et al. 2007). Questionnaires on teacher strengths and difficulties were completed for only 48.4% of the study population. Most of the children were not assessed because either their teachers were absent or the information was not available at the time of the researchers going to the school. It is essential to have dependable data on the numbers of children enrolled in schools. Generally, the student population in the schools was dynamic, with some transferring between schools, dropouts and class repeats. This volatility had implications on school registers, which did not always reflect current information. During data collection activities, this study also faced challenges with coordinating all study undertakings in such a way that interferences to teaching and school activities were minimised. In spite of these issues, the study was undertaken and demonstrated that research in these settings can be conducted in a practical and feasible manner that is acceptable to the local communities.

## Limitations

- The research method employed in this current study was exploratory and descriptive. When using exploratory research, it is not possible to predict or explain behaviour by manipulating or measuring different variables. This cross-sectional study could not assess trends over time or the temporal sequence between exposures such as anaemia and iron deficiency and outcome variables such as growth, development and school performance. As a result, conclusions regarding causal relationships could not be made. However, it is important to point out that it was never the intention of this study to make cause-and-effect conclusions regarding the developmental progression of anaemic and non-anaemic children but merely to examine and describe their developmental profiles and determine whether any differences exist. Therefore, with regard to this study objective, this limitation was contained.
- Because of the cross-sectional study design, only the prevalence and not the incidence was measured; hence, prevalence–incidence bias cannot be ruled out because long-standing cases of anaemia and iron deficiency may be over-represented while short-lived cases may be under-represented.
- The current exposure and outcome variables were assessed simultaneously, which could result in the overlooking of recent changes in anaemia or iron status in children with identified impairments. The proportion of children with resolved impairments, anaemia or iron deficiency could not be determined because this study did not test for evidence of past disease.
- The small sample size was not adequate for the analysis of some outcome variables; hence, conclusions drawn from these analyses could be erroneous given the lack of power to detect correlations for some of the outcomes.

## Recommendations

The present study needs to be replicated with a larger, more diverse population sample with countrywide involvement. A large-scale analysis could provide valuable information on child growth and development that may be generalised to the country’s child population. A larger sample would also reduce the chance of bias. To realise a measurable effect of anaemia and iron status on early learning, it may be necessary to modify research approaches so as to assess changes in health status of school-aged children over time with implementation of interventions for anaemia control and educational quality. Unresolved questions that persist include establishing the relative effect of acute and chronic iron deficiency as well as considering the importance of the severity of iron deficiency. The importance of a marginal intake of iron is unclear, where reserves are depleted although anaemia has not developed. The quality of evidence relating to how anaemia or iron deficiency affects cognitive abilities or the impact of NIDA on intellect is poor. This topic has been subject to relatively little examination and needs further exploration.

## Conclusion

The findings from this study show the complexities of health status and educational achievement in rural schools during the foundation school learning stages. This study has presented findings that show that child health, particularly anaemia and iron status, may impact child growth, development and school performance. Nonetheless, the impact on school performance did not reach statistical significance. No significant differences were observed in psychomotor development and school performance of the children based on their anaemia and iron status or between children with IDA and NIDA. Possible reasons for these outcomes could be that all the sampled children had inadequate learning resources, including schoolbooks or quality teaching. The children in this study sample were attending poorly resourced schools that were historically black African schools. The children in this sample population had 1 or 2 years’ schooling, which may have been insufficient. A prolonged period of teaching to allow for effective learning before the evaluation may have been beneficial. A similar study with a larger sample size may also improve the statistical power and enable definitive conclusions to be drawn about the relationship between anaemia and iron deficiency and the assessed outcome variables.
